# Therapeutic Potential of Human Amniotic Epithelial Cells on Injuries and Disorders in the Central Nervous System

**DOI:** 10.1155/2019/5432301

**Published:** 2019-11-20

**Authors:** Huiming Xu, Jiaofei Zhang, Kam Sze Tsang, Hao Yang, Wei-Qiang Gao

**Affiliations:** ^1^State Key Laboratory of Oncogenes and Related Genes, Renji-Med-X Clinical Stem Cell Research Center, Renji Hospital, School of Medicine, Shanghai Jiao Tong University, Shanghai 200127, China; ^2^Prince of Wales Hospital, Division of Neurosurgery, Department of Surgery, Chinese University of Hong Kong, Hong Kong SAR, China; ^3^Translational Medicine Center, Honghui Hospital, Xian Jiao Tong University, Xian 710054, China; ^4^Med-X Research Institute and School of Biomedical Engineering, Shanghai Jiao Tong University, Shanghai 200030, China

## Abstract

Despite recent advances in neurosurgery and pharmaceuticals, contemporary treatments are ineffective in restoring lost neurological functions in patients with injuries and disorders of the central nervous system (CNS). Therefore, novel and effective therapies are urgently needed. Recent studies have indicated that stem cells, including embryonic stem cells (ESCs), induced pluripotent stem cells (iPSCs), and mesenchymal stem cells (MSCs), could repair/replace damaged or degenerative neurons and improve functional recovery in both preclinical and clinical trials. However, there are many unanswered questions and unsolved issues regarding stem cell therapy in terms of potency, stability, oncogenicity, immune response, cell sources, and ethics. Currently, human amniotic epithelial cells (hAECs) derived from the amnion exhibit considerable advantages over other stem cells and have drawn much attention from researchers. hAECs are readily available, pose no ethical concerns, and have little risk of tumorigenicity and immunogenicity. Mounting evidence has shown that hAECs can promote neural cell survival and regeneration, repair affected neurons, and reestablish damaged neural connections. It is suggested that hAECs may be the most promising candidate for cell-based therapy of neurological diseases. In this review, we mainly focus on recent advances and potential applications of hAECs for treating various CNS injuries and neurodegenerative disorders. We also discuss current hurdles and challenges regarding hAEC therapies.

## 1. Introduction

Central nervous system (CNS) injuries and disorders seriously affect human health and quality of life. Hitherto, neurosurgery and pharmaceutical agents can alleviate symptoms, but no effective therapy is available to repair/replace damaged or degenerated neurons and restore neurological functions [1]. The identification of novel and effective treatment modalities is crucial. Currently, stem cell therapy has drawn much attention as a promising therapeutic option for the treatment of various neurological diseases. Various stem cells, including embryonic stem cells (ESCs), induced pluripotent stem cells (iPSCs), neural stem cells (NSCs), and mesenchymal stem cells (MSCs), have been investigated for their therapeutic potential in the treatment of neurological disorders in preclinical and clinical trials. In addition, studies have shown that stem cells can increase neurological recovery, allowing reconnections of disrupted neural circuits [2, 3].

Previous studies have indicated that different types of stem cells dictate and inherit unique lineage-specific characteristics, leading to a diverse extent of cellular functions (proliferation, differentiation, immunogenicity, and tumorigenicity) [4–6], but they might also excite clinical and ethical unease if concerns are not addressed and properly resolved prior to translation from bench to bedside. Among these stem cells, MSCs derived from umbilical cord blood, bone marrow, and adipose tissues have been studied in clinical trials for neurological diseases and have been shown to exert neuroprotective effects [7]. However, cell resources, invasive extraction procedures, and cell quantity make this type of stem cell less favourable as a practical source for cell therapy. Human umbilical cord-derived MSCs (UCMSCs) have been used in clinical trials as a treatment for some neurological diseases since 2011. Currently, 24 registered studies of UCMSCs have been listed at http://ClincalTrials.gov, and some trials have been completed. However, only one clinical trial has reported that UCMSCs are safe and might delay the procession of Hereditary Spinocerebellar Ataxia [8]. Therefore, the safety and efficacy of UCMSC therapy for neurological diseases require further assessments in clinical trials. Recently, preclinical studies have suggested that human amniotic epithelial cells (hAECs) derived from the human amnion might be a better alternative cell source for CNS injuries and diseases as they are readily available, have no tumorigenic and low immunogenic potential, are under less ethical dispute, and are efficient in the treatment of CNS injuries and diseases [9–15]. In this review, we mainly focus on hAECs and summarize the advances regarding hAEC-based therapies in preclinical studies of neurological injuries and neurodegenerative diseases, including the possible mechanisms following treatment with hAECs (as summarized in Table 1).

### 1.1. Characteristics of hAECs

**Figure 1:**
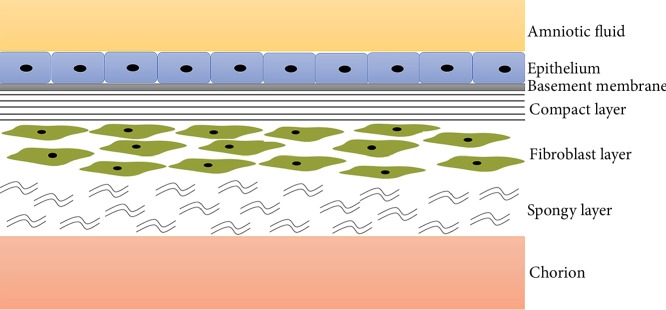
Schematic diagram of the amnion. The amnion consists of five histological layers, namely, epithelial monolayer, basement membrane, a compact layer, a fibroblast layer, and an intermediate or spongy layer.

hAECs are derived from the epithelial layer of the amnion, which is the membranous sac enclosing the foetus and amniotic fluid that protects the developing embryo against various stimuli from the surroundings [16]. The amnion is a translucent biological membrane lacking nerves, muscles, and lymphatic vessels and consists of five different layers (Figure 1) [17]. Beneath the epithelial layers, there are a compact stromal layer and a fibroblast layer, from which amniotic mesenchymal stem cells (AMSCs) are derived. AMSCs exert a neuroprotective function in ischaemic animal models [18]; however, AMSCs have not been extensively studied in other neurological disease models. In this review, we mainly focus on the potential therapy of hAECs in the treatment of neurological diseases.

Notably, hAECs possess substantial advantages over the other stem cells. hAECs are derived from term placenta, which is discarded after birth. Thus, they are easily available, require no invasive procedures for harvesting, and lack any relevant ethical issues. Furthermore, hAECs have low expressions of HLA-A, B, and C and HLA-DR, which are key antigens in recipient rejection [19], and express the nonpolymorphic, nonclassic antigen HLA-G, which can directly suppress immune responses, suggesting that hAECs are of weak immunogenicity [20]. A recent study has also demonstrated that intravenous administration of hAECs does not result in haemolysis, allergic reactions, toxicity or tumour formation, indicating that hAECs are systematically safe [21]. No signs of acute rejection have been noted in an early clinical trial of hAECs in a small cohort of seven subjects up to seven weeks postimplantation [19]. The bioactivities of cells and tissues derived from the human amniotic membrane have long been used in patients suffering from skin burns or ocular burns, also suggesting that hAECs do not induce recipient immune rejection [22, 23]. The human amniotic membrane originates from pluripotent epiblasts prior to gastrulation; it is likely that hAECs possess multilineage differentiation potential and can therefore differentiate into all three germ layers, including neural cells [24]. This is in agreement with the findings that hAECs are able to differentiate into neuronal-like cells *in vivo* [9, 14, 15, 25]. In addition, it has also been reported that hAECs are able to synthesize and release neurotrophic factors (NTFs), growth factors, and neurotransmitters such as catecholamine and dopamine, which promote neural survival and regeneration and exhibit multiple neuronal functions [26–29]. Therefore, it is reasonable to believe that hAECs may be a potential cell source for cell-based therapy of neurological diseases.

## 2. Prospective Applications of hAECs

### 2.1. Stroke

Stroke is one of the leading causes of death and disability worldwide. Effective therapy is currently unavailable. In the past few decades, stem cell therapy has been actively explored in the treatment of stroke. Data show that stem cells can reduce the size of infarcts and improve functional recovery by promoting survival and regeneration of neurons and repairing damaged brain tissue [30]. Among different stem cell sources, bone marrow MSCs (BM-MSCs) are widely studied in clinical trials. Autologous BM-MSCs can be expanded *ex vivo*, but it is extremely difficult for patients to obtain cells in predetermined doses in time. On the other hand, readily available frozen stocks of allogeneic hAECs are an alternative stem cell source.

Regarding the treatment of ischemic stroke using hAECs, one previous study reported that hAECs were transplanted by intracerebral (i.c.) injection into rats subjected to transient middle cerebral artery occlusion (MCAO). The grafts reduced the infarct volume and cerebral apoptosis and improved motor and cognitive functions 16 days poststroke. Moreover, transplanted hAECs were noted to express neuronal markers, neuronal progenitor markers, and astrocyte markers, which suggested that hAECs could transform into neuronal-like cells and could contribute to the repair of affected neurons [9]. Despite the beneficial outcomes of hAEC-based therapy for ischemic stroke, i.c. injection is not practical for several reasons. First, i.c. injections require expensive imaging equipment and surgical expertise. Further, i.c. injection may cause additional brain injury and induce a heightened inflammatory response within the brain, and the approach is unlikely to target the systemic immunosuppression effects of stroke [31]. Recently, Evans et al. explored the efficacy of systemically delivered hAECs in ischemic stroke animal models. They found that hAECs administered by intravenous (i.v.) injection to stroke mice at the acute phase and subacute phase could attenuate behavioural deficits and did not cause immune rejection. When administered to mice acutely after stroke (1.5 hours poststroke), hAECs could migrate to the infarct area, reduce the infarct size, attenuate the infiltration of immune cells, and modulate inflammatory responses. It was noted that a considerable number of hAECs migrated to the spleen, which may effectively attenuate poststroke systemic immunosuppression and be beneficial for overall recovery and the brain repair process [32]. Systemic immunosuppression, including splenocyte apoptosis, splenic atrophy, loss of splenic and circulating leukocytes, and a weakened type 1 T-helper response, likely contributes to lung infection, which is a major cause of poststroke mortality and morbidity [33]. When mice were administered with hAECs 1–3 days poststroke, long-term functional recovery was observed, and more intact neural cells were evident in the peri-infarct cortex [32]. Moreover, neuroprotection of hAECs was also observed in a marmoset monkey stroke model [32, 34]. Preclinical data suggest that administration of hAECs by i.v. injection during the acute and subacute phases of stroke might be safe and effective for the repair and recovery of neurological function.

In haemorrhagic brain injury, activation of microglia occurs shortly after intracerebral hemorrhage (ICH). Accumulating data indicate that activated microglial cells lead to secondary brain injuries, including inflammation, which increases the permeability of the blood-brain barrier (BBB) and ultimately causes brain edema and neuronal death [35]. In ICH animal models, hAEC grafts could enhance neural cell survival and regeneration in the perifocal tissue [14, 25]. Moreover, activated microglia were suppressed in the perihematoma regions, and inflammatory factor levels of TNF-*α*, IL-1*β*, and MMP-12 were reduced, which might be attributed to the reduced extent of brain edema and neurological deficits [14, 36]. Taken together, preclinical studies suggest that hAEC therapy may be effective in the treatment of ischemic stroke and ICH by the following potential mechanism. First, hAECs could differentiate into neural tissue to replace damaged or dead neural cells. Second, hAECs could suppress the inflammatory response by inhibiting the activation of microglial cells and producing anti-inflammatory factors and immunosuppressive factors, which contribute to the protection of neurons from immune cell-mediated apoptosis. Third, hAECs could secrete necessary cytokines, NTFs, and growth factors, which provide a favourable microenvironment for the survival and regeneration of neural cells and synaptogenesis, eventually contributing to the reinnervation of lost connections and restoring cellular function [36–40]. Finally, systemically administered hAECs could attenuate poststroke immunosuppression, attributable to the lower extent of infection and beneficial for overall recovery and brain repair processes.

### 2.2. Spinal Cord Injury (SCI)

SCI is a severe debilitating disease that usually accompanies motor and sensory dysfunctions [41]. Contemporary medical interventions focus on stabilizing the spine and controlling inflammation to prevent further damage. Currently, there is no effective treatment modality available for the recovery of this type of neurological function. Among possible new strategies, stem cell transplantation is a promising treatment for SCI. Stem cells can repair damaged neural cells and allow axonal regrowth, resulting in the reestablishment of neural circuits and functional recovery and brain repair processes.

Sankar and Muthusamy reported that hAECs grafted into a hemisection cavity promoted the growth of axotomized axons and prevented the formation of glial scars at the transection lesion site [42]. Consistent with this study, Wu et al. found that hAECs could promote axon regeneration and sprouting, inhibit the atrophy of axotomized red nuclei, and improve hindlimb function in SCI rats [10]. Moreover, hAEC grafts could also alleviate SCI-induced neuropathic pain [43]. In addition, coimplantation of hAECs with NSCs into SCI rats could enhance the survival of host neurons and promote the survival and neuronal differentiation of transplanted NSCs. A significant improvement in behavior was observed in the SCI rats receiving hAECs and NSCs [44]. Notably, a bridging strategy that allows an axon to grow across the lesion site is beneficial to spinal cord repair [45]. Transplantation of hAECs seeded on acellular muscle scaffolds or silk fibres into spinal cord hemisectioned rats could significantly promote axonal growth and remyelinate nerve fibres, leading to motor function recovery of SCI rats [46, 47]. These preclinical studies indicate that hAECs can allow regrowth of damaged axons by inhibiting the activation of microglial cells and the formation of glial scars and producing paracrine factors to optimize spinal cord microenvironments, resulting in the reestablishment of damaged neural connections and functional recovery. Moreover, the strategies of hAEC-embedded biomaterials and cotransplantation of hAECs and NSCs might be good options to facilitate the treatment of SCI.

### 2.3. Cerebral Palsy (CP)

CP is a common neurodevelopmental disorder of preterm and term infants. Cerebral white matter (WM) injury, known as periventricular leukomalacia (PVL), is a predominant neuropathology associated with CP [48]. Two key pathways that contribute to neonatal WM injury are abnormal neonatal cerebral haemodynamics and localized cerebral inflammation. The underlying mechanism, including activated microglia, astrocyte proliferation, increased permeability of the BBB, and oligodendrocyte death, can result in WM injury and subsequently influence brain development [49, 50]. Currently, there is no treatment for this injury.

hAECs have been shown to have strong immunomodulatory abilities by reducing microglia activation and producing anti-inflammatory and immunosuppressive factors, suggesting that hAECs may be a potential therapy for WM injury [36, 37, 39, 40]. In intrauterine inflammation animal models, cerebral WM injury was induced by lipopolysaccharides (LPS) or high tidal volume (*V*_*T*_) mechanical ventilation, and hAECs were intravenously transplanted into foetal tissue. hAECs were found to migrate to multiple regions of the brain and reduce the inflammatory response and neural injury in the foetal brain, as evidenced by a decrease in the numbers of activated microglia and in the permeability of the BBB. Significantly, more oligodendrocytes and neurons appeared in the subcortical and periventricular WM, implying that hAECs prevent WM injuries in preterm foetal brains [51–53]. In addition, oxidative stress can also increase the probability of developing a WM injury in preterm or term infants [54, 55]. To address this problem, a neonatal inflammation and perinatal hyperoxia mouse model was used. hAEC administration rescued the decreased body weight and reduced apoptosis and astrocyte areal coverage in the WM [56]. Data suggest that hAECs protected WM development in the preterm and term infant brain by reducing the cerebral inflammatory response and producing paracrine factors attributed to the regeneration of oligodendrocytes and neurons in the subcortical and periventricular WM.

### 2.4. Parkinson's Disease (PD)

PD is a neurodegenerative disorder characterized by a progressive loss of dopaminergic neurons in the substantia nigra, cytoplasmic aggregated Lewy bodies, and neuroinflammation. Its typical symptoms mainly include resting tremors, muscle rigidity, slowness, and gait abnormalities [57]. Despite the advent of pharmaceutics and neurosurgery, PD symptoms can only be relieved. Curative treatment for PD is not yet available.

Regarding hAEC-based therapies for PD, Kakishita et al. reported that hAECs transplanted to the striatum of PD rats could relieve their behavioural deficits. Engrafted hAECs were noted to not only express tyrosine hydroxylase but also prevent degeneration of nigral dopaminergic neurons [15, 58]. Consistent with these studies, Yang et al. also demonstrated the effects of hAECs on PD rats through their neurodifferentiation and neuroprotection [11, 59]. In addition, we found that engrafted hAECs could reduce microglial activation and inflammatory factor levels in PD mice (unpublished data). In summary, hAEC-based therapy for PD mainly relies on its neurogenic potential, anti-inflammatory effects, and the ability to synthesize and release NTFs and neurotransmitters [26–28, 39, 40].

### 2.5. Alzheimer's Disease (AD)

AD is an irreversible, progressive neurodegenerative disease. Its neuropathology is characterized by the aggregation of extracellular beta-amyloid into plaques, intracellular neurofibrillary tangles with abnormally phosphorylated tau proteins and inflammation, and deficits in the cholinergic system associated with a decrease in acetylcholine activity [60, 61]. AD is also regarded as the major cause of dementia [62]. Conventional therapy is based on the inhibition of acetylcholinesterase, which can only delay the progression of mental deterioration and reduce neuropsychiatric symptoms but cannot cure AD [63]. Repopulation and reestablishment of lost neuronal connections or circuits by stem cell transplantation might be a potential treatment modality [64].

Xue et al. demonstrated that hAECs transplanted into the lateral ventricles of APP/PS1 AD mice could increase the numbers of hippocampal cholinergic neurons and acetylcholine concentration in the hippocampus of experimental mice. Moreover, mice with engrafted hAECs displayed a significant improvement in spatial memory [12, 65]. Preclinical data suggest that the therapeutic benefit of hAECs in the treatment of AD might mainly rely on paracrine factors that promote the survival and regeneration of cholinergic neurons, eventually leading to the improvement of spatial memory.

### 2.6. Multiple Sclerosis (MS)

MS is a chronic inflammatory demyelinating disease of the CNS, characterized by the inflammation and destruction of the myelin sheaths of neurons, resulting in a disruption in the communication of different parts of the nervous system with each other **[**66**]**. There are many novel pharmaceutical compounds beneficial to MS with anti-inflammatory, remyelinating, and neuroprotective effects; however, severe adverse effects are prominent [67]. It is noteworthy that hAECs can secrete many NTFs [26], which can stimulate remyelination and protect oligodendrocytes against apoptosis in order to restore and maintain neurological function in MS [68–70]. hAECs were shown to produce anti-inflammatory factors and immunosuppressive factors, which are strong immunomodulators [36–40]. Therefore, hAECs could be a promising cell source for the treatment of MS.

Liu et al. intravenously injected hAECs into experimental autoimmune encephalomyelitis (EAE) mice, which is an animal model used to study the pathogenesis of MS. hAECs ameliorated relapse and remission and significantly reduced demyelination in EAE mice [13, 71]. In addition, hAECs may exert immunomodulatory effects in EAE mice, as evidenced by an increase in the numbers of anti-inflammatory Th2 cells and Tregs, the maintenance of the peripheral naïve CD4^+^ T cell pool [13, 71], and the suppression of pathogenic T cell responses in peripheral lymphoid organs and within the CNS of EAE mice [71, 72]. Moreover, levels of IL-2, IL-5, and IL-10 were increased after hAEC treatment, which represents a change towards a beneficial cytokine profile [13, 71]. Preclinical studies suggest that hAECs may hold a promise in clinical trials for treating MS regarding their beneficial effects of immunomodulation, neuroprotection, and remyelination.

## 3. Clinical Trials

Human amniotic membranes have long been used for the treatment of a variety of injuries and diseases, including acute corneal injuries, skin burns, and diabetic foot ulcers [22, 73–75]. Recently, hAECs derived from human amnions have also drawn much attention. Currently, 10 registered studies of hAECs are listed at http://ClincalTrials.gov, with one trial investigating CNS disease. In addition, there are four phase I trials of hAECs registered in the Australian New Zealand Clinical Trials Registry at http://www.anzctr.org.au: ACTRN12618000076279 for ischaemic stroke [76], ACTRN12614000174684 and ACTRN12618000920291 for bronchopulmonary dysplasia [77], and ACTRN12616000437460 for liver cirrhosis [78]. Phase I clinical hAEC therapy for ischemic stroke was designed to determine the maximal tolerated dose (MTD) and assess cell safety. Fifteen stroke patients were recruited and injected with hAECs by intravenous infusion. The final follow-up of 15 patients in the hAEC arm has not been completed until now. Safety and efficacy will be assessed by the frequency of SAEs, imaging, and immunological assays [76]. Therefore, more trials are required to assess and determine the safety and clinical benefits of hAEC-based therapy.

## 4. Current Challenges for hAEC-Based Therapy

For a safe perspective, we know that hAECs did not induce immune rejection or tumour formation from an early clinical trial of hAECs in a small cohort of seven healthy humans up to seven weeks postimplantation [19]. Furthermore, from 1981 up to now, the frequency of SAEs in the seven volunteers participating in the clinical trial has not been reported. Indeed, the bioactivities of amnion cells and tissues have long been exploited to treat skin burns or ocular burns [22, 23]. Therefore, it is reasonably predicted that hAECs can be safely administered to patients. Additionally, data from preclinical studies suggest that hAEC-based therapies could be promising for neurological injuries and diseases. However, there are still some challenges in the implementation of hAEC therapy. First, hAECs are isolated from the epithelial layer of the amnion, expressing high levels of Epcam (90%). Among these cells, some are CD90- (mesenchymal marker) positive and others are CD90-negative cells (named CD90^+^ Epcam^+^ and CD90^−^ Epcam^+^). In some studies, hAECs did not include CD90^+^ Epcam^+^ cells [76, 79]. However, another study showed that CD90^+^ Epcam^+^ cells possessed a more vigorous immunoregulatory ability [21]. Whether CD90^+^ Epcam^+^ and CD90^−^ Epcam^+^ cell subtypes have different effects on neurological diseases remains to be investigated. In addition, hAECs at passage 0 (P0) and P5 have different expression profiles of surface markers [79]. Which passage of hAECs is suitable for clinical use still needs to be determined. Thus, the activity, potency, and purity of hAECs must be verified and validated for their release for clinical evaluation [21, 79]. Furthermore, a comprehensive knowledge of how transplanted hAECs exert their therapeutic effects is not yet fully understood. Certainly, more clinical trials for different types and stages of neurological diseases in larger cohorts of patients for long-term monitoring are desirable to attest the clinical relevance of hAECs.

The goal of stem cell therapies is amenable for replenishing and reestablishing lost neural connections [2, 3]; thus, systematic controls of secondary injuries attributed to neurotoxic microenvironments are important to maintain the survival and functions of hAECs at sites adjacent to the lesion areas of the parenchyma [80, 81]. Apart from cell quantity, host factors (subtype of the disease, different stages of the disease, and lesion location), therapeutic time window (acute, subacute, or chronic), delivery route (intracerebral, intravenous, or intra-arterial), and outcome measures (behavioural outcomes and imaging assessment) also have a substantial impact on the success of hAEC therapies [9, 32, 76, 82–86].

It has been reported that cotransplantation of hAECs with NSCs or the forced overexpression of trophic factors in these cells could strengthen the beneficial effects of hAECs on neurological diseases [9, 44]. hAECs embedded in biomaterial scaffolds support cell survival and differentiation after implantation and provide a good microenvironment for nerve regeneration and functional recovery [47]. More *in vivo* and long-term preclinical studies are needed before the translation from bench to bedside can occur.

## 5. Conclusions

**Figure 2:**
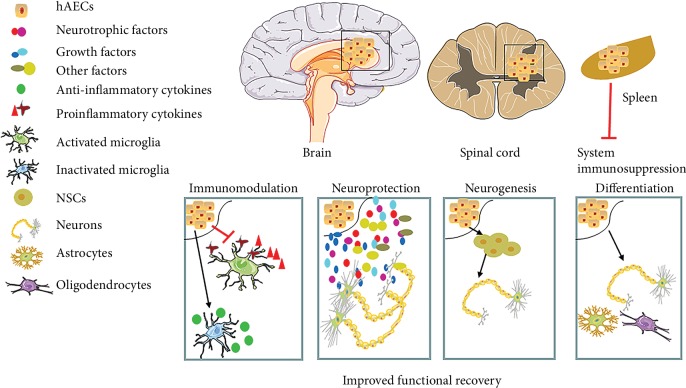
Human amniotic epithelial cell- (hAEC-) based therapy for neurological injuries and diseases. After hAECs are transplanted into the brain by intracerebral or intravenous injection, hEACs migrate to the lesion area of the brain, and the likely therapeutic mechanisms of hAECs in the treatment of neurological injuries and diseases are listed.

Increasing evidence in the literature suggests that stem cell therapy is amenable to diseases and disorders related to cell loss and degeneration in the CNS. To date, among various stem cell sources, hAECs appear to be an ideal candidate for cell therapy since hAECs are readily available, have no tumorigenic and low immunogenic potential, and are less ethically disputable compared to other stem cell sources. There are several potential therapeutic mechanisms of hAECs in the treatment of neurological injuries and diseases, as shown in Figure 2. First, hAECs possess the neurogenetic potential to differentiate into neural cell types. Second, via paracrine mechanisms, hAECs can secrete many necessary cytokines, NTFs, growth factors, hormones, and/or neurotransmitters to facilitate neural survival and regeneration, axonal outgrowth, and synapse reformation, thus leading to the reinnervation of lost neuronal connections and further recovery of neurological functions. In particular, exosomes, 30-150 nm extracellular vesicles, including proteins, DNA fragments, phospholipids, and RNAs, mediate various biological functions, such as immune responses, antigen presentation, intercellular communication, protein, and RNA transfer. Exosomes also play an important role in the nervous system including neuronal development, regeneration, synaptic function, and functional recovery in neurological diseases [87, 88]. Recently, exosomes isolated from conditioned media of hAECs (hAEC Exo) prevented bleomycin-induced lung injury in young and aged mice and exerted antifibrotic, immunomodulatory, and regenerative properties. Some specific proteins and miRNAs rich in the cargo of hAEC Exo might be essential for the immunomodulation and the anti-fibrotic and stem cell pluripotent pathway [89]. Moreover, hAEC Exo can accelerate wound healing, inhibit scar formation [90], and restore ovarian function by miRNAs against apoptosis [91]. Of course, the clinical application of hAEC Exo for neurological diseases requires further investigation. Third, hAECs may contribute to endogenous neurogenesis and functional recovery as they can enhance neuronal differentiation of NSCs [44, 92]. Fourth, hAECs can modulate the immune response and reduce inflammatory responses, protecting neuronal cells from apoptosis and contributing to neural recovery. Currently, an increasing body of literature indicates that stem cells exert neuroprotective effects that are more likely attributed to their paracrine effects and anti-inflammatory responses [87, 88, 93, 94]. Thus, hAECs may play neuroprotective roles by a similar mechanism. More studies are needed to define the mechanism of hAECs in the treatment of neurological diseases.

## Figures and Tables

**Table 1:** hAECs administered in animal models of injuries and diseases of the CNS and the possible mechanisms.

Diseases	Authors	Model animal	Delivery route	Suggested mechanism	Clinical improvement
Stroke	[9]	Acute ischemia rats	I.C.	Reduced apoptosis Neural differentiation	Reduced infarct volume Improved functional behaviour
Stroke	[32] [34]	Acute ischemia mice and marmoset monkeys	I.V.	Reduced cerebral apoptosis and inflammation Reduced systemic immunosuppression	Reduced infarct volume Improved functional behaviour
Stroke	[14]	ICH rats	Intracerebral	Reduced microglial activation Increased neural cell survival and regeneration	Reduced brain edema Ameliorated neurologic deficits
Stroke	[25]	ICH rabbits	Intracerebral	Neural differentiation	Improved functional behaviour
Stroke	[36]	ICH rats	Intracerebral	Reduced microglial activation and inflammatory factors	Reduced inflammation response
SCI	[42]	SCI monkeys	Injected into the transection cavities	Promote the growth of axotomized axons Prevent the formation of glial scar	Prevented the formation of glial scar Prevented death in axotomized cells
SCI	[10]	SCI rats	Injected into spinal transections	Promote the regeneration of the axons Inhibited the atrophy of axotomized red nucleus	Improved functional behaviour
SCI	[43]	SCI rats	Injected into spinal transections	Reduced microglial activation	Alleviated SCI-induced mechanical allodynia
SCI	[44]	SCI rats	Transplanted into spinal transections	Enhanced the survival of host neurons Promoted the survival and neural differentiation of codrafted NSCs	Improved functional behaviour
SCI	[46]	SCI rats	Transplanted into spinal hemisections with muscle scaffold	Promoted axonal growth Promoted the remyelination of nerve fibres	Improved functional recovery
SCI	[47]	SCI rats	Transplanted into spinal hemisections with silk fibroin scaffold	Reduced the formation of glial scar Reduced immunological reaction at lesion site	Improved functional behaviour
Parkinson's	[15]	PD rats	Intracerebral	Promoted the differentiation of the neurons expressing tyrosine hydroxylase	Improved functional behaviour
Parkinson's	[58]	PD rats	Intracerebral	Promoted the survival of dopamine neurons	Improved the survival of dopamine neurons
Parkinson's	[11]	PD rats	Intracerebroventricular	Prevented the loss of dopamine neurons and their metabolites	Improved behaviour recovery
Parkinson's	[59]	PD rats	Intracerebral	Increased the content of dopamine neurons and their metabolites	Improved behaviour deficits
Alzheimer's	[12] [65]	APP/PS1 mice	Intracerebral	Increased survival of cholinergic neurons Promoted acetylcholine production	Improved spatial memory
MS	[13]	EAE mice	Intravenous	Suppressed the proliferation of splenocytes and T cells Increased proportion of Th2 production	Reduced demyelination Ameliorated relapse and remission
MS	[71]	EAE mice	Intravenous	Inhibited the proliferation of splenocytes Increased the production of IL-2 and IL-5	Decreased remission Demyelination
MS	[72]	EAE mice	Intravenous	Increased the number of Treg and naïve CD4^+^ T cells Decreased the T cell response and inflammatory factors	Ameliorated relapse
CP	[52]	Brain injury in preterm foetal sheep	Foetal jugular vein	Reduced microglial activation, apoptosis and astrogliosis, and BBB integrity maintenance	Protected the developing brain
CP	[51]	Brain injury in preterm foetal sheep	Foetal brachial artery	Reduced microglial activation, pyknotic cells, and cell death Increased oligodendrocytes and MBP-positive cells	Restored myelin morphology Decreased white matter injury
CP	[53]	White matter injury in preterm foetal sheep	Intravenous and intratracheal	Reduced microglial activation and vascular leakage	Modulated white matter pathology
CP	[56]	Brain injury in perinatal mouse	Intravenous	Reduced apoptosis and astrogliosis Increased microglial activity	Rescued the decreased body weight
